# Fabrication of Silicon Carbide Nanoparticles Using Pulsed Laser Ablation in Liquid and Viscosity Optimization via Solvent Tuning

**DOI:** 10.3390/ma17184527

**Published:** 2024-09-14

**Authors:** Saeid Heidarinassab, Anesu Nyabadza, Inam Ul Ahad, Dermot Brabazon

**Affiliations:** 1 I-From, Advanced Manufacturing Research Centre, Advanced Processing Technology Research Centre, School of Mechanical and Manufacturing Engineering, Dublin City University, D09 V209 Dublin, Ireland; 2 EPSRC & SFI Centre for Doctoral Training (CDT) in Advanced Metallic Systems, School of Mechanical and Manufacturing Engineering, Dublin City University, D09 V209 Dublin, Ireland

**Keywords:** nanoparticles, laser, synthesis, silicon, PLAL

## Abstract

In this study, silicon carbide nanoparticles (NPs) were produced via pulsed laser ablation in liquid, aiming to investigate the influence of processing parameters on the properties of the resultant NPs and their applicability for inkjet printing. The results revealed an increase in NP concentration with increasing laser power, but the maximal absorbance in the case of 0.743 and 1.505 W is lower than that for 1.282 W laser. Dynamic light scattering was employed to determine the size distribution of the NPs, demonstrating a range of 89 to 155 nm in diameter. Notably, an inverse relationship was established between increasing laser scanning speed and pulse repetition rate (PRR) and the mean size of the NPs. Higher PRR and laser power exhibited an augmentation in the concentration of NPs. Conversely, an increase in scanning speed resulted in a reduction in NP concentration. Based on FTIR, data formation of SiC NPs based on the target material is the most dominant behavior observed followed by an amount of oxidation of the NPs. Examination of the resulting NPs through field emission scanning electron microscopy equipped with energy-dispersive X-ray analysis (EDX) unveiled a predominantly spherical morphology, accompanied by particle agglomeration in some cases, and the elemental composition showed silicon, carbon, and some oxygen present in the resulting NPs. Furthermore, the modulation of colloidal solution viscosity was explored by incorporating glycerol, yielding a maximal viscosity of 10.95 mPa·s.

## 1. Introduction

Pulsed laser ablation in liquid (PLAL) is a technique used to create nanoparticles (NPs) by directing laser energy onto a target submerged in a liquid. This process generates NPs using the material from the target, offering a top-down approach to nanoparticle synthesis [1,2,3,4]. In recent years, there has been a growing fascination with employing PLAL to produce colloids for diverse applications. This method operates within a simple system maintained at room temperature, is considered a green way of NP fabrication, and reduces thermal damage, which are the advantages associated with this method rather than other NP fabrication methods. It can be applied either statically or dynamically. While the dynamic mode has shown superior productivity compared to the static mode, the latter offers the advantage of simplicity in obtaining colloidal NPs. PLAL has been extensively utilized for synthesizing various metallic NPs [3,4]. The technique has been used for synthesizing different metallic NPs including magnesium [5,6], carbon [7,8], gold [9], silver [10,11], silicon [12], and ceramic NPs including silicon carbide [12,13,14], titanium oxide [15], and silver oxide [11]. A highly notable aspect of NPs is their optical properties, which are heavily influenced by both their size and shape [16]. Indeed, PLAL presents a promising avenue for nanoparticle production based on bulk target materials. It offers several advantages over other nanoparticle production methods. Firstly, it is easily accessible for atmospheric ablation, eliminating the need for a specialized environment such as a vacuum. Additionally, the method is simple and does not require complex setups. Moreover, the properties and morphology of the NPs can be tailored by adjusting laser parameters and liquid media, enhancing their versatility and applicability in various fields [15,17]. In PLAL, various parameters such as laser fluence, pulse repetition rate (PRR), scanning speed, ablation time, and the choice of liquid media play crucial roles in determining the physical and chemical properties of the produced NPs. Adjusting these parameters allows for precise control over the size, shape, and composition of the NPs synthesized. For instance, organic solvents were used for the PLAL, and their effect was studied on the resulting NPs [18]. Moreover, PLAL has been adapted into different formats, including laser-assisted etching and magnetic field-assisted PLAL. These adaptations have enabled the production of a wide range of metallic NPs with tailored properties, expanding the applicability of the technique across different fields and applications [19,20]. The productivity of the PLAL process has become a focal point in current research within the field. Studies, such as those by Wagner and Barcikowski et al. [21], have shed light on specific factors influencing productivity. For instance, they found that the pulse repetition rate significantly impacts the ablation efficiency of zinc/zinc oxide. Moreover, post-irradiation processes have been identified as crucial in determining nanoparticle productivity. For instance, research has demonstrated that the overall productivity of platinum NPs decreases as the focal point of the laser increases. These findings underscore the intricate interplay of various parameters in optimizing the productivity of PLAL and highlight avenues for further exploration and enhancement of the technique [22]. Indeed, the study suggests that post-irradiation of the laser leads to fragmentation, influencing both the productivity and size of the NPs produced. This finding highlights the complexity of the PLAL process and the need for further investigation into optimizing productivity. The ongoing exploration of PLAL productivity underscores its significance in nanoparticle synthesis and its potential for various applications, driving continued interest and research in the field. For instance, Dittrich et al. [23] suggested that the most productive laser among those studied is the high-power picosecond laser, exhibiting a 12-fold increase in ablation rate when compared to ablation in liquid. Additionally, it is noted that the threshold fluence required for ablation in air is up to 1.9 times higher than that for ablation in water. Interestingly, the most efficient ablation, defined as the energy-specific ablation volume divided by laser power, is achieved by the low-power and compact nanosecond laser system.

Expanding the narrative, research on PLAL has extended to explore its application for specific materials like silicon carbide (SiC), which boasts exceptional properties crucial for high-temperature applications. SiC NPs find extensive use in various applications requiring elevated temperatures and high frequencies, such as light-emitting diodes and UV detectors. Moreover, SiC exhibits excellent high-temperature properties, including oxidation resistance, high thermal conductivity, and a low thermal expansion coefficient. These qualities make it a valuable raw material for advanced refractories, functional ceramics, and abrasives. The utilization of PLAL for synthesizing SiC NPs opens up avenues for leveraging its unique properties across diverse industrial and technological domains, further driving the exploration and advancement of this nanoparticle synthesis technique [13,24,25]. Indeed, several methods exist for producing silicon carbide (SiC) NPs, with pulsed laser ablation in liquid (PLAL) notably emerging as a prominent technique in recent years. PLAL offers distinct advantages over other methods, driving its increasing adoption in SiC nanoparticle synthesis. These benefits include the ability to produce NPs without the need for extreme conditions such as high temperatures or pressures, and the capacity to tailor nanoparticle properties through adjustments in laser parameters and liquid media composition. The growing prominence of PLAL in SiC nanoparticle synthesis reflects its effectiveness and versatility in meeting the demands of various applications requiring SiC NPs with specific characteristics. For instance, Castelleto et al. [26] fabricated 4H-SiC NPs using ultra-short pulsed laser ablation for biomedical imaging applications, whereas Yamada et al. [27] studied the resulting sizes, shapes, and crystal structure of produced SiC NPs using femtosecond PLAL in acetone. Few data have been reported on the pulsed laser ablation in liquid of SiC NPs in DI water using a laser source, which requires further investigation to understand the correlation of the PLAL parameters and SiC NP properties [28,29,30,31].

The literature on silicon carbide (SiC) nanoparticle production via pulsed laser ablation in liquid (PLAL) for various applications is extensive. However, a notable gap exists regarding studies that explore the correlation between the properties of the resulting NPs and the adjustment of colloidal viscosity using different solvents for inkjet printing applications. This gap presents an opportunity for research to investigate the optimization of colloidal viscosity as ink for inkjet printing applications, particularly considering the promising potential of inkjet printing SiC NPs for high-temperature industries, leveraging SiC’s high thermal conductivity. This paper aims to address this gap by focusing on the fabrication of nanocolloids containing SiC NPs and optimizing the colloidal viscosity for inkjet printing applications. Additionally, it seeks to establish a connection between the characteristics of SiC NPs synthesized through PLAL and the effect of laser processing parameters on the resulting NPs’ properties. By filling this gap, this study could contribute valuable insights into enhancing the applicability of SiC NPs in inkjet printing for high-temperature industries.

## 2. Experimental

### 2.1. NP Synthesis

The silicon carbide rod was purchased from GoodFellow (Cambridge UK) and used as the target in the ablation process. The rod was divided into 50 sections, each 5 mm in height and 5 mm in diameter. Before each laser ablation procedure, the target underwent polishing using a 2000-grit-sized polishing paper and was then positioned vertically in a 100 mL glass beaker. Utilizing Design Expert software version 13, a 3 × 3 full factorial experimental design (as depicted in Table 1) was created to explore three different laser powers (0.743 W, 1.282 W, and 1.504 W), pulse repetition rates (PRRs) of 10 kHz, 30 kHz, and 50 kHz, and scanning speeds of 1 m/s, 2 m/s, and 3 m/s, resulting in a total of 27 samples. Parameters such as ablation time, laser spot diameter, working distance, and liquid media remained constant at 5 min, 50 µm, 34.03 mm, and de-ionized water, respectively. A Nd:YAG laser system (WEDGE HF 1064, Bright Solutions Pavia, Italy) emitting pulses centered at 1064 nm with a pulse width of 600 ps and a repetition rate of 10 kHz was employed for the ablation process. The target was submerged in 10 mL of de-ionized water, maintaining a distance of 5 mm between the water surface and the target. Laser ablation was performed using an Archimedean spiral pattern scanning strategy with an outer diameter of 2 mm and a hatch spacing of 50 µm.

### 2.2. NP Characterization

Ultraviolet–visible spectroscopy (UV-Vis) measurements were conducted to determine the absorbance of SiC NPs as an indicator of NP yield. The UV-Vis analysis was performed using a Shimadzu UV-2600 UV-Vis spectrometer (Kyoto, Japan) equipped with a quartz cuvette (10 mm pathlength) within a scan range of 185–300 nm. Prior to measurements, a baseline scan was conducted to ensure the proper functioning of the UV-Vis absorbance detector and optimal conditions within the cuvette. All optical spectra were corrected for liquid medium absorption by subtracting its contribution from the recorded spectrum. Additionally, the mean sizes and concentrations of NPs (particles per mL) were determined using dynamic light scattering (DLS) with a Malvern Zetasizer Ultra. Three measurements were taken for each sample and averaged. The absorbance coefficient and refractive index values of SiC were incorporated as input parameters for DLS analysis. Furthermore, Fourier-transform infrared spectroscopy (FTIR) was employed using a PerkinElmer Spectrum Two FT-IR instrument to investigate the bonds present in the synthesized NPs and their structural characteristics. FTIR scans were conducted within the range of 4000–400 cm^−1^ after drop-casting the synthesized NPs onto a glass substrate and allowing them to dry. A background scan was conducted using FTIR in which the glass substrate was placed in the instrument. The spectral peaks associated with the glass substrate were subsequently subtracted from the recorded spectrum to ensure accurate analysis. pH measurements of both de-ionized water and resulting colloids were performed using a pH meter (Orion 420A+ Thermo Electron Corporation, Waltham, MA, USA). For morphological analysis, field emission scanning electron microscopy (FESEM) was carried out using a Hitachi S5500 Field Emission SEM (Ibaraki, Japan). A drop (0.1 µL) of SiC colloid was deposited onto a 400-mesh size copper grid, followed by air pumping for 2 min to facilitate water evaporation (drop-casting) before SEM examination. The STEM sample holder was utilized to secure the grid, and careful handling with smooth tweezers was employed to avoid surface damage. Energy Dispersive X-ray analysis (EDX) was utilized in conjunction with FESEM to examine the composition of the colloid drop and map its constituents. Colloidal viscosity was measured using a viscometer (Brookfield Ametek DVNext Cone/Plate Rheometer, Harlow, UK) with a rotational speed of 60 RPM at 25 °C for 5 min. Calibration of the instrument was conducted before measuring each sample’s viscosity. Initially, viscosity measurements were taken without any additives, followed by measurements after adding three different additives to the colloids: acetone (99.9% purity), isopropyl alcohol (99.9% purity), and glycerol solution (86% purity). Viscosity measurements were performed three times for each sample and averaged.

## 3. Results and Discussion

### 3.1. Visual Inspection and Ultraviolet–Visible Spectroscopy

**Figure 1:**
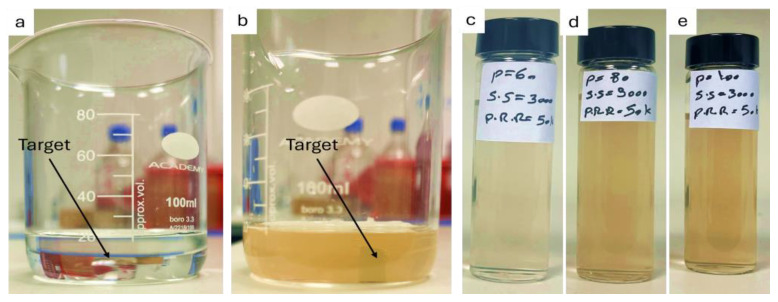
Photographs of (**a**) DI water with SiC target before PLAL and (**b**) after 5 min PLA; a set of samples produced at a constant PRR of 50 kHz; a scanning speed of 3 m/s; and laser powers of (**c**) 0.743, (**d**) 1.282, and (**e**) 1.504 W.

Figure 1a depicts a beaker containing deionized (DI) water with the SiC sample target, while Figure 1b illustrates the same beaker with the target after 5 min of laser ablation. In Figure 1b, the color of the liquid medium has transitioned from colorless to brown following the 5 min laser ablation process. This observed change in color has been previously documented in the existing literature [26], where the color transition is attributed to the presence of SiC NPs within the liquid medium. In Figure 1c–e, a series of samples are presented, prepared with a constant pulse repetition rate (PRR) of 50 kHz and varying laser powers with different scanning speeds. Upon increasing the laser power from 0.743 W to 1.504 W, a subtle change in color intensity from light brown to dark brown was observed. This shift in color before and after PLAL is attributed to the presence of SiC NPs, with the slight intensity change in the brown color correlating with increased NP yield across the liquids [26]. Therefore, visual inspection indicated that increasing the laser power and scanning speed resulted in an increase in the NP yield.

**Figure 2:**
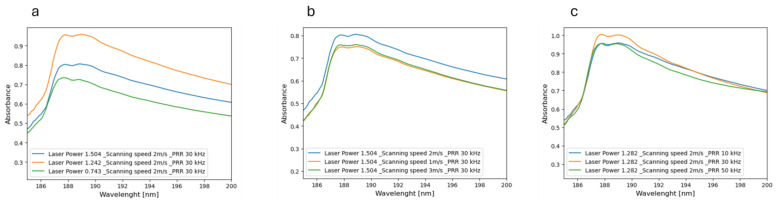
UV-Vis measurements for the samples with varying laser processing parameters of (**a**) laser power, (**b**) scanning speed, and (**c**) pulse repletion rate (PRR). In each graph two other variables are kept constant and one variable changes.

Additionally, UV-Vis analysis was conducted on each sample post-experimentation to validate the presence of SiC NPs, with the corresponding extinction spectra depicted in Figure 2. To isolate the contribution of SiC NPs, the pattern of deionized (DI) water was subtracted from the overall spectrum. This was achieved by introducing a reference sample of DI water into the instrument while recording the colloid spectrum. The recorded spectra for all produced samples are provided in Figure S1 in the supplementary data. In Figure 2a–c, extinction spectra for the produced colloid samples were categorized based on the same laser power, with varying scanning speeds and pulse repetition rates (PRRs) indicated from left to right (laser powers of 0.743 W and 1.282 W, scanning speeds ‘ss’ between 1, 2, and 3 m/s, and PRRs of 10, 30, and 50 kHz, respectively). Generally, all recorded spectra exhibited a consistent trend with a peak at 195 nm, which can be associated with the formation of polyynes during the synthesis of carbon NPs in DI water [32]. This peak also has been reported in a previous work [12]. Indeed, it is important to acknowledge the possibility of silicon atoms reacting with water to form silicon oxide, which could contribute to the observed phenomena. Additionally, as noted, increasing the laser power led to a rise in absorbance (including scattering) from 0.743 to 1.505 W, attributed to the increased concentration of NPs within the liquid. This can be attributed to the higher energy provided by the increased laser power, facilitating greater vaporization or ablation of the target material and consequently, the production of more NPs [33]. Interestingly, the absorbance of resulting NPs produced at a laser power of 1.282 W was notably higher compared to other laser powers. Moreover, at higher wavelengths up to 300 nm, the absorbance of resulting SiC NPs across all laser powers exhibited a linear decline, reaching a saturation point. The pH of the deionized water was initially measured at 7.63, and subsequent measurements of the resulting samples from the PLAL process yielded pH values ranging from 7.63 to 7.22. This consistent range suggests minimal pH alteration during the production of silicon carbide (SiC) via PLAL.

**Figure 3:**
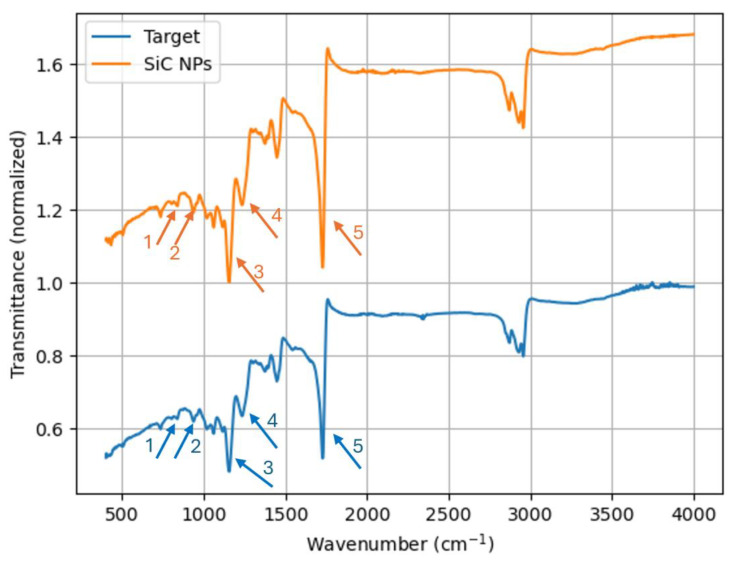
FTIR spectra of the target material and the produced NPs.

Furthermore, both the target material and the synthesized NPs were analyzed using an FTIR spectrometer, with the corresponding spectra displayed in Figure 3. Table 2 represents the peaks observed in the FTIR spectrum with their associated bond and materials. These spectra prominently feature a band at 1080 cm^−1^, accompanied by a shoulder at 1140 cm^−1^, and another band at 1769 cm^−1^. The doublet band is associated with the asymmetric Si-O-Si stretch, while the band at 1769 cm^−1^ corresponds to the C=O stretch. Additionally, a smaller peak observed at 880 cm^−1^ is attributed to the Si-O-Si bending mode [34] or Si-O-Si vibration in a (SiO)_n_ ring configuration [35]. It is also reported to be longitudinal and transverse TO and LO phonons of the SiC lattice [36]. The peak at 840 cm^−1^ resembles very much the absorption of nano-SiC which is also observed in previous work [37]. Therefore, this peak is certainly assigned to the formation of SiC. The group of weak bands between 2000 and 3000 cm^−1^ can be correlated to C-H bending at 2000 and stretching at 2830. The observed bands of the target material are the same bands for the produced NPs, suggesting the structure of the NPs is the same as the target material.

### 3.2. Dynamic Light Scattering (DLS)

DLS measurements were utilized to assess the average size and quantity of NPs produced through PLAL across varying scanning speeds, PRRs, and laser powers. Supplementary data include the recorded DLS spectra for all samples in Figure 2.

**Figure 4:**
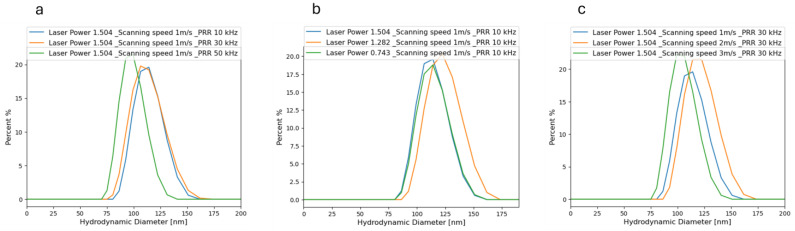
DLS mass-weighted hydrodynamic diameters measurement for the samples with varying laser processing parameters of (**a**) PRR, (**b**) laser power, and (**c**) scanning speed. In each graph, two other variables were kept constant, and one variable changed.

In general, as evident in Figure 4, NPs exhibited mean sizes ranging from 89 to 155 nm. Figure 4 also showed a narrower size range observed for samples generated at laser powers of 1.282 W and 1.504 W compared to those at 0.743 W.

**Figure 5:**
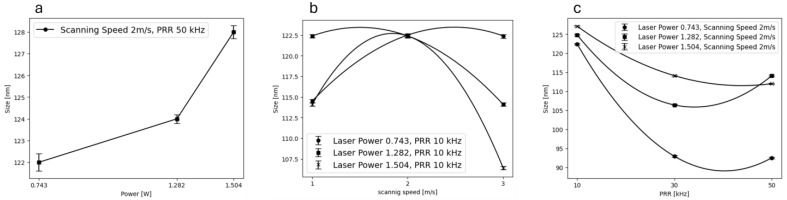
The DLS data illustrate the change in NPs’ mean diameter sizes with changing (**a**) laser power, (**b**) scanning speed, and (**c**) PRR.

Figure 5 illustrates how adjustments in laser parameters impact the mean sizes of NPs. Notably, a rise in scanning speed from 1 to 3 m/s resulted in a consistent decrease in mean size. This phenomenon is attributed to the shift from sporadic to consistent ablation processes. Figure 5a–c demonstrate the alterations in NPs’ mean sizes corresponding to changes in laser parameters. Specifically, Figure 5a illustrates the variations in mean size with increasing scanning speed at a constant PRR of 10 kHz and laser powers of 0.743 W, 1.282 W, and 1.505 W. As depicted in Figure 5a, a consistent reduction in mean size is observed with the increment in scanning speed from 1 to 3 m/s. This phenomenon can be attributed to the intermittent ablation process under low scanning speeds, characterized by the repetitive modulation of the plume shielding effect impacting particle ejection and morphological characteristics. Conversely, at higher scanning speeds, the ablation process reaches a steady state as the plume shielding effect is overcome [38]. In Figure 5b, the fluctuations in NPs’ mean sizes are depicted in response to changes in the pulse repetition rate (PRR), while maintaining a constant laser power of 0.743 W, 1.282 W, and 1.505 W, and a scanning speed of 2 m/s. The overall trend suggests a reduction in nanoparticle size with increasing PRRs. This pattern can be attributed to the elevated heating of the target material resulting from the cumulative impact of multiple laser pulses at higher repetition rates. Such increased thermal effects are likely to influence the size and morphology of the generated NPs, potentially leading to the formation of smaller particles [39]. Furthermore, by increasing the PRR up to 50 kHz, the NP sizes declined for all the resulting NPs produced in various laser powers. Moreover, Figure 5c shows the changing of the NPs’ size with changing laser powers at a constant PRR of 10 kHz and a scanning speed of 2 m/s. Based on this figure, the size of NPs exhibited an increase with rising laser power. Specifically, the sizes were measured at 122 nm, 124 nm, and 128 nm for samples produced at laser powers of 0.743 W, 1.282 W, and 1.505 W, respectively. This observed trend indicates that higher laser power resulted in the production of larger NPs. The relationship between laser power and nanoparticle size can be attributed to its direct influence on the thermodynamic properties of cavitation bubbles and the resulting plasma. Additionally, higher laser power contributes to increased local concentration of ablated species due to liquid confinement. This facilitates the plasma plume to attain higher temperature and pressure, consequently prolonging the duration of cavitation bubbles. Such conditions are conducive to the formation of larger-sized particles through coalescence [40]. Another factor contributing to this observation could be the formation of bubbles in the liquid at the applied laser power. These bubbles create a discontinuity in the refractive index at the interfaces between the liquid and gas phases, resulting in significant light scattering by the laser. This scattering phenomenon diminishes the amount of laser energy that reaches the solid target, consequently reducing the total quantity of NPs generated in the liquid.

**Figure 6:**
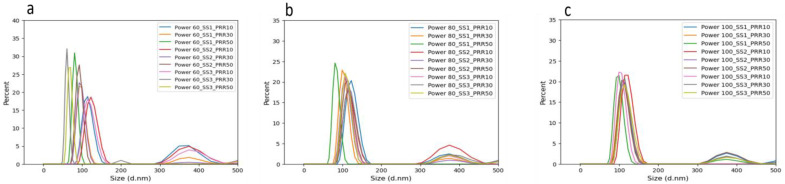
Dynamic light scattering mass-weighted hydrodynamic diameter patterns in a size range of up to 450 nm for a set of samples with laser powers of (**a**) 0.743 W, (**b**) 1.282, and (**c**) 1.505 W.

Furthermore, as illustrated in Figure 6a–c, a size range of 410 to 450 nm was consistently observed across all samples. This size range can be attributed to either the agglomeration of NPs within the colloids or the direct ejection of SiC from the target material.

**Figure 7:**
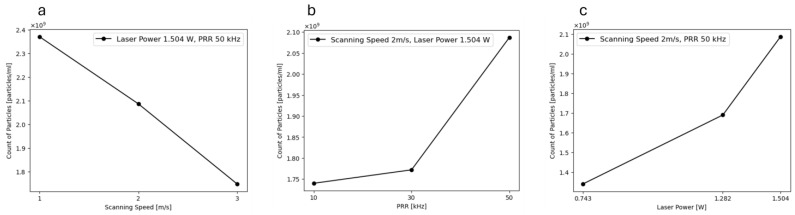
The NPs’ concentration measurement as a function of (**a**) scanning speed, (**b**) PRR, and (**c**) laser power.

Nanoparticle concentration measurements were conducted, and the results are presented in Figure 7a–c. In each graph, two variables were held constant to evaluate the impact of changing one parameter on particle concentration. The concentration measurements were performed on the same cuvette containing aggregated particles. Based on these findings, the concentration of NPs increased from 1.71 × 10^9^ to 2.07 × 10^9^ particles/mL with an increase in PRR from 10 to 50 kHz. Similarly, an increase in laser power from 0.743 W to 1.505 W led to an increase in nanoparticle concentration from 1.32 × 10^9^ to 2.1 × 10^9^ particles/mL. This observation aligns well with the visual inspection results, where an increase in nanoparticle yield was observed with higher laser power. Conversely, increasing the scanning speed resulted in a decrease in nanoparticle concentration. Additionally, a broader size distribution was observed at lower scanning speeds, possibly due to the fragmentation of particles [41]. It is also worth mentioning that, after releasing the NPs into the liquid medium, they may further oxidize and slowly grow to a limited extent, or they may agglomerate [42,43]. In this case, since no stabilizer was used, the stability of the NPs is low, and therefore, agglomeration is expected to occur. The concentration of NPs in a liquid under pulsed laser ablation is influenced by the scanning speed. In general, lower scanning speeds tend to result in a higher concentration of nanoparticles, whereas higher scanning speeds lead to a lower concentration. This phenomenon is attributed to the residence time of the laser beam on the target material, which is influenced by the scanning speed. Increasing the scanning speed reduces the number of laser pulses at any given point on the target and increases the distance between consecutive pulses. Consequently, higher scanning rates lead to a decrease in the number of pulses impacting a specific area of the target material [44], which reduces material ablation and NP generation. Lower scanning speeds, on the other hand, enable the laser pulses to remain on the target material for a longer period of time, resulting in greater ablation and NP production [45].

### 3.3. Field Emission Scanning Electron Microscopy (FESEM) and Scanning Transmission Electron Microscopy (STEM)

**Figure 8:**
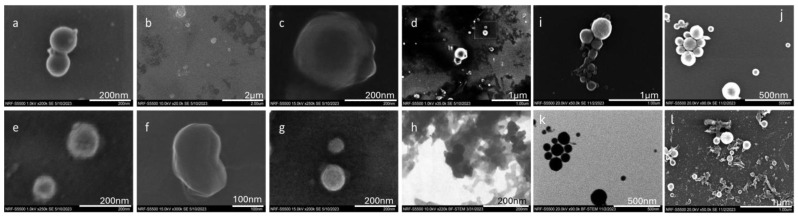
FESEM images of produced SiC NPs: (**a**,**d**,**e**) SE acceleration voltage 1 kV, (**c**,**f**) SE acceleration voltage 15 kV, (**b**,**g**) SE acceleration voltage 10 kV, (**i**,**j**,**l**) SE acceleration voltage 20 kV, and (**h**,**k**) bright-field STEM acceleration voltage 10 kV and 20 kV, respectively.

FESEM was employed to capture images of the resulting nanoparticle shapes. The colloids obtained were drop-cast onto a 400-mesh copper grid and air-dried for 5 min to evaporate the colloid water. Figure 8a–g depict the FESEM images, while Figure 8h presents the bright-field scanning transmission electron microscopy (STEM) image of dried SiC NPs generated at a laser power of 0.743 W, a scanning speed of 2 m/s, and a PRR of 10 kHz, showcasing different acceleration voltages and magnifications. These figures confirm the presence of spherical and nanoscale NPs with some agglomerations and clusters, as observed in the STEM image. The primary mechanism underlying picosecond and nanosecond pulsed laser ablation is thermal evaporation. In this process, the laser generates high temperatures on the surfaces of larger particles, causing them to melt and vaporize into atoms or molecules. Subsequently, these atoms reassemble into smaller nanostructures with similar or different shapes, depending on the surrounding medium and laser parameters [46]. As the surrounding medium in this case is DI water, all the NPs have the same shape which is a mixture of spherical and non-spherical shapes with some agglomeration resulting in the formation of clusters. Moreover, the dipole moment of the liquid medium significantly influences the shape of the resulting NPs [47]. When growth is dominated by the nucleation of atoms in the condensed phase in a constant dipole moment of the liquid medium, spherical particles are likely to be formed. In this case, as the dipole moment of DI water (1.85D) remains consistent across all produced NPs, the shape of the NPs should be similar throughout all the produced samples. Furthermore, as evident from Figure 8a,b,d,f,h, the observed agglomeration in NPs aligns well with the DLS findings (refer to Figure 6), where a peak was identified in the size range of 410 to 450 nm. Additionally, the rapid expansion of vapor bubbles in the superheated liquid led to the forceful ejection of the target material in particulate form, which may serve as another contributing factor to the generation of large-sized particles [48].

**Figure 9:**
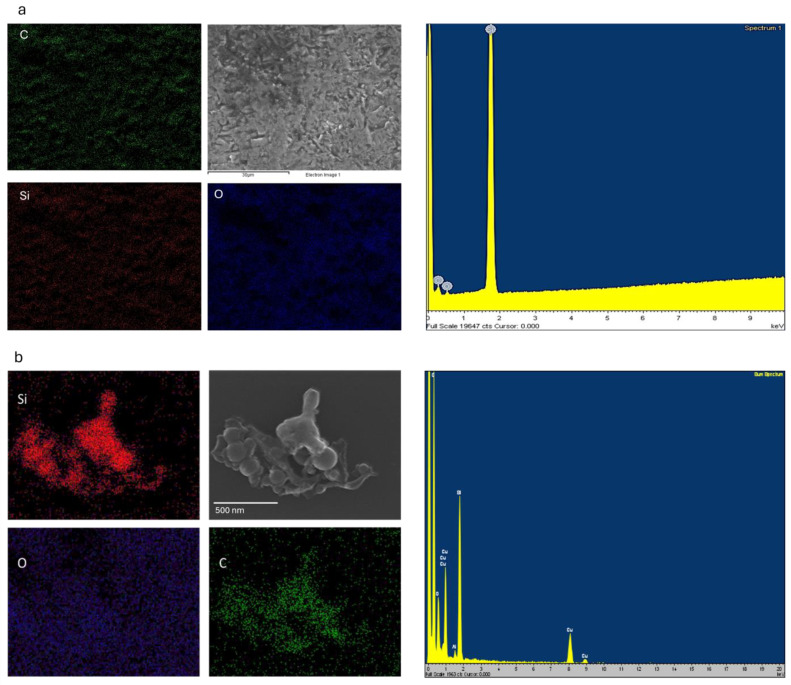
The EDX result and elemental mapping of the (**a**) SiC target material and (**b**) resulting NPs.

**Figure 10:**
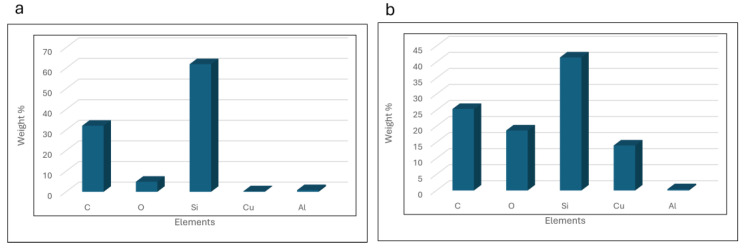
Weight percentages of the elements observed in the EDX for the (**a**) target material and (**b**) produced NPs.

In addition, Figure 9a and Figure 9b illustrate the EDX result of the target material and the resulting NPs, respectively. Generally, the produced NPs are the same material as the target material. As obvious from Figure 8b, carbon is the most dominant element in the NPs followed by silicon and oxygen. The elemental mapping images clearly confirm that the NPs observed consist of carbon and silicon. There is the amount of copper recorded in the EDX results which comes from the copper grid used for the FESEM, followed by a small amount of aluminum which is associated with contamination in the chamber or the sample preparation process. There is also oxygen present in the EDX result which further confirms the oxidation of the NPs during the PLAL. Table 3 shows the elemental weight percentage based on the obtained result in EDX. Figure 10 presents the weight percentages of elements in both the target material and the fabricated NPs, as determined by Energy Dispersive X-ray (EDX) analysis. The data in Figure 10 reveal a notable increase in the amounts of carbon, silicon, and oxygen in the fabricated NPs compared to the target material. The elevated silicon and carbon content in the NPs indicates the successful formation of silicon carbide, with a silicon-to-carbon ratio of approximately 1.5:1. Moreover, the oxidation level was calculated based on the EDX results where a silicon-to-oxygen ratio of 2.3:1 was achieved. Additionally, the increased weight percentage of oxygen in the fabricated NPs suggests the presence of silicon oxide on their surface. Based on EDX results and the FTIR data, the primary observation is the formation of silicon carbide (SiC) nanoparticles from the target material. Additionally, a minor degree of oxidation of the nanoparticles is also detected.

### 3.4. Viscosity Optimization and Effect of Liquid Media

**Figure 11:**
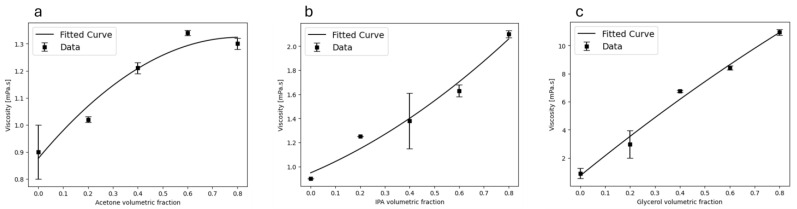
Dynamic viscosity measurements at 25 °C as a function of the volumetric concentration of (**a**) acetone, (**b**) IPA, and (**c**) glycerol.

These NPs, devoid of any additives, hold promise for application in ink production for inkjet printing purposes. The ink’s fluid characteristics, encompassing viscosity, particle size, particle concentration, density, and surface tension, play pivotal roles in the printing process. Maintaining appropriate ink viscosity, typically ranging from 1 to 20 mPa.s, is essential to avoid complications such as excessive flow or blockage of the printer head [32]. In this study, the viscosity of the nanocolloids containing SiC NPs was adjusted to meet the viscosity criteria suitable for commercially available inkjet printers by incorporating various solvents. Specifically, three solvents—acetone, isopropyl alcohol (IPA), and glycerol—were blended with the colloids in varying volumetric ratios to modulate the viscosity of the pure NP colloids. The viscosity values as a function of solvent volumetric ratios are illustrated in Figure 11a–c and summarized in Table 4 for acetone, IPA, and glycerol, respectively. A viscosity range of 0.9 to 1.41 mPa.s was achieved, with the maximum viscosity observed at a 0.7 volumetric fraction of acetone in water (Figure 11c). Furthermore, a maximum viscosity of 10.95 mPa.s was attained at a 0.8 volumetric fraction of glycerol in water. For the IPA–water mixture, a viscosity range of 0.9 to 2.34 mPa.s was obtained, with the maximum viscosity recorded at a 0.8 volumetric fraction of the acetone–water mixture. These findings are consistent with previous research [32]. Several factors, including the interaction between NPs and the solvent, can cause changes in the viscosity of the liquid. Glycerol, a non-toxic and highly viscous solvent, was used in varying proportions with water to achieve a wide range of viscosity values.

Furthermore, the liquid medium can not only affect the viscosity but also influence the oxidation of particles in long-term storage. While oxidation itself does not directly impact the printability of particles, it can significantly alter their properties after inkjet printing, such as thermal and electrical conductivity [6,49,50,51]. Studies have shown that liquids like water cause more oxidation compared to organic solvents such as IPA and acetone. This is due to water’s higher oxygen content (33 atomic %) compared to IPA (8.3 atomic %) and acetone (10 atomic %), its higher reactivity, and its ability to readily form hydrogen bonds. Increased oxidation is associated with reduced thermal and electrical conductivity because oxide layers are thermal insulators and hinder charge mobility. Moreover, oxide layer formation on nanoparticles over time is an indication of instability, as it can lead to the formation of larger particles or agglomerates due to a decrease in Zeta potentials over time. These agglomerates can subsequently cause issues such as print head clogging during inkjet printing especially if the colloids are left unused for a long period after synthesis (e.g., a month). Additionally, organic solvents tend to have lower boiling points than water, making them easier to inkjet print without the need to heat the print bed to high temperatures. This can be advantageous in processes that require rapid drying or when printing on heat-sensitive substrates such as low-melting-point polymers. On the other hand, water is abundant and inexpensive, which makes it an attractive option for the mass production of colloids, despite the potential challenges associated with its higher boiling point and tendency to cause oxidation.

The applicability of PLAL in inkjet printing is highly promising due to its ability to precisely tailor nanocolloid properties, including nanoparticle size, concentration, stability, and colloid viscosity, as demonstrated herein. The future of PLAL appears to lie in its flow mode application [52,53,54], which incorporates automated inline quality control through UV-Vis and DLS measurements. In this setup, nanocolloids are produced continuously and characterized in real time for quality control, enabling the mass production of colloids. According to Freeland et al. [55,56], flow mode PLAL with real-time, closed-loop process monitoring offers three key advantages for scalability and sustainability. First, it increases throughput via automation, continuous nanocolloid generation, and the avoidance of laser shielding effects. Second, it provides robust quality control, ensuring that colloids meet inkjet printing specifications before being collected from the PLAL process. Third, it reduces waste through the closed-loop control system. Many review papers have highlighted PLAL as one of the most sustainable methods for producing nanocolloids for inkjet printing [2,4,57,58,59]. This is largely due to the minimal waste generated, the lack of need for high-temperature reactors as required in other thermophysical methods, the absence of harsh chemicals common in wet chemistry techniques, and the reduced necessity for impeccable pH control compared to biological methods. Generally, PLAL requires minimal post-processing, unlike chemical methods where particles need to be cleaned of ligands and acids, or biological methods where nanoparticles must be separated from the biological organisms used in synthesis (e.g., bacteria or fungi). This reduction in material, processing time, and energy requirements enhances the sustainability of PLAL compared to the biological or chemical methods currently employed in the industry.

## 4. Conclusions

This study investigated the application of pulsed laser ablation in liquid for producing silicon carbide NPs suitable for inkjet printing applications. The influence of PLAL parameters such as laser power, scanning speed, and pulse repetition rate on the properties of the resulting NPs—including yield, optical characteristics, size, and concentration—was thoroughly examined.

The findings revealed that increasing laser power correlated with higher NP yield across all tested liquids. Moreover, the optical absorbance increased with laser power, attributed to higher NP concentration within the liquid. A distinct peak at a wavelength of 195 nm indicated the formation of polyynes.

FTIR analysis highlighted the predominant formation of SiC NPs from the target material, with some level of oxidation observed. DLS results indicated a mean NP size distribution ranging from 89 to 155 nm, with smaller sizes observed at higher scanning speeds and pulse repetition rates.

Concentration measurements demonstrated that increasing PRR and laser power led to higher NP concentrations, while higher scanning speeds resulted in lower concentrations. FESEM imaging revealed the production of spherical NPs with consistent sizes.

Finally, viscosity measurements were conducted to tailor the colloids for inkjet printing. A glycerol–water mixture with a volumetric fraction of 0.8 yielded a maximum viscosity of 10.95 mPa.s, suitable for processing with commercial inkjet printers.

In conclusion, PLAL offers a cost-effective approach for producing SiC NP colloids with adjustable viscosities, presenting a promising avenue for SiC colloid production tailored for inkjet printing applications. As observed in this study, oxidation has occurred on the surface of the resulting NPs and this phenomenon has to be taken into account for accurate inkjet printing applications.

## Figures and Tables

**Table 1:** Design of experiments used for production of sample colloids.

Level	−1	0	1
Laser power (W)	0.743	1.282	1.504
Scanning speed (m/s)	1	2	3
PRR (kHz)	10	30	50

**Table 2:** Derived peak position from FTIR spectrum with their associated material.

Peak Number	Bonding	Associated Material
1	Si-C	Silicon Carbide
2	Si-O-Si	Silicon Oxide/TO and LO Scratching of SiC Lattice
3	Si-O-Si	Silicon Oxide
4	Si-O-Si	Silicon Oxide
5	C=O	Carbonyl Compound or Carbide

**Table 3:** The elements and their weight percentages in both the target and the fabricated NPs based on EDX results.

Element	Weight Percentage (Target)	Weight Percentage (NPs)
Carbon	32.06	25.4
Silicon	61.95	41.46
Oxygen	4.89	18.71
Copper	0.27	14.03
Aluminium	0.83	0.4

**Table 4:** The viscosity of the colloids using a 0.8 volumetric fraction of acetone, IPA, and glycerol.

Solvent	Viscosity (mPa·s)
Acetone	0.95
IPA	2.93
Glycerol	10.95

## Data Availability

The original contributions presented in the study are included in the article/supplementary material, further inquiries can be directed to the corresponding author.

## Supplementary Materials

The following supporting information can be downloaded at: https://www.mdpi.com/article/10.3390/ma17184527/s1, Figure S1. UV-Vis measurement for all the produced samples categorized based on a constant laser power of (a) 0.743, (b) 1.282, and (c) 1.504 W; Figure S2. DLS measurement for all the produced samples categorized based on a constant laser power of (a) 0.743, (b) 1.282, and (c) 1.504 W.
