# Supplementary material for: Fabrication of Silicon Carbide Nanoparticles Using Pulsed Laser Ablation in Liquid and Viscosity Optimization via Solvent Tuning
Source: Materials (Basel). 2024 Sep 14;17(18):4527. doi: 10.3390/ma17184527 (PMC11433422; doi:10.3390/ma17184527)
Supplement: Supplementary file 1 [file materials-17-04527-s001.zip › materials-3105405-supplementary.pdf]

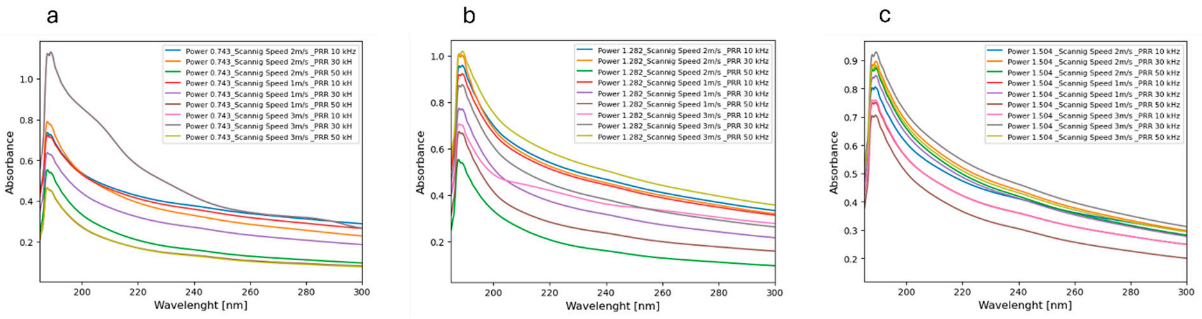

Figure S1. UV-Vis measurement for all the produced samples categorized based on a constant laser power of a) 0.743, b)1.282, and c)1.504 W

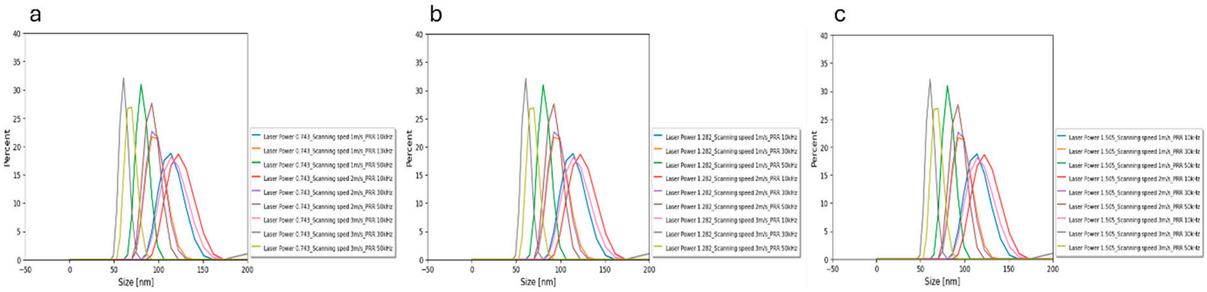

Figure S2. DLS measurement for all the produced samples categorized based on a constant laser power of a)0.743, b)1.282, and c)1.504 W
